# Brain lateralization probed by water diffusion at the atomic to micrometric scale

**DOI:** 10.1038/s41598-019-51022-1

**Published:** 2019-10-11

**Authors:** F. Natali, C. Dolce, J. Peters, C. Stelletta, B. Demé, J. Ollivier, G. Leduc, A. Cupane, E. L. Barbier

**Affiliations:** 1 Institut Laue-Langevin, 71 avenue des Martyrs, CS 20156, 38042 Grenoble cedex 9, France; 2 CNR-IOM, OGG, 71 avenue des Martyrs, CS 20156, 38042 Grenoble cedex 9, France; 3 University Grenoble Alpes, LiPhy, 140 rue de la physique, 38402 Saint Martin d’Hères, France; 4 Department of Physics and Chemistry, University of Palermo, via Archirafi 36, 90123 Palermo, Italy; 5 Department of Animal Med., Production and Health, University of Padova, Viale dell’Università 16, 35020 Agripolis, Legnaro Italy; 6 Biomedical Facility, ESRF, 71 avenue des Martyrs, CS 20156, 38042 Grenoble cedex 9, France; 7 University Grenoble Alpes, Inserm, U1216, Grenoble Institut Neurosciences, 38000 Grenoble, France

**Keywords:** Molecular biophysics, Molecular biophysics, Biological physics, Biological physics

## Abstract

Combined neutron scattering and diffusion nuclear magnetic resonance experiments have been used to reveal significant interregional asymmetries (lateralization) in bovine brain hemispheres in terms of myelin arrangement and water dynamics at micron to atomic scales. Thicker myelin sheaths were found in the left hemisphere using neutron diffraction. 4.7 T *d*MRI and quasi-elastic neutron experiments highlighted significant differences in the properties of water dynamics in the two hemispheres. The results were interpreted in terms of hemisphere-dependent cellular composition (number of neurons, cell distribution, etc.) as well as specificity of neurological functions (such as preferential networking).

## Introduction

An important topic in the study of the neural basis of brain functions is the interregional asymmetry between the left and the right hemisphere and its relation to the factors that modulate cognitive specialization in the brain, such as language and motor control. The specialization of the two hemispheres is termed lateralization.

The central nervous system (CNS) comprises the brain (cerebrum and cerebellum) and the spinal cord^1^. The brain contains neurons that receive, analyze and store information. It is also the source of conscious and unconscious thoughts and behaviors. The cerebrum is one of the most important parts of the brain; it controls emotion, hearing, vision, personality, amongst other things. The cerebrum, which accounts for 85% of total brain weight, is divided into left and right hemispheres. The hemispheres communicate with each other through the corpus callosum (a bundle of fibers between the hemispheres). Of particular interest is the modularity of the hemispheres, which allows one hemisphere to take over a specific function controlled by the other hemisphere if the latter is damaged. It should be noted, however, that this ability depends on the area damaged and the patient’s age. Generally speaking, the left hemisphere controls the right side of the body and vice versa. Typically, in humans, the left hemisphere handles linear reasoning and language functions, such as vocabulary and grammar, while the right hemisphere accounts for different language functions, such as intonation and accentuations, as well as for the processing of visual and audiological stimuli, spatial manipulation, facial perception, and artistic ability^2–4^. Other integrative functions, including arithmetic, sound localization and emotions, seem to be controlled more bilaterally^5,6^.

The networks in the human right hemisphere are anatomically more efficient and interconnected than those in the left hemisphere^7^. In terms of functional principles, these patterns appear to support the fact that the left hemisphere plays a leading role in highly demanding specific processes, such as language and motor actions, which may require specialized networks, whereas the right hemisphere is responsible for more general processes, such as integration tasks, which may require a more general level of interconnection. The connectivity of a given region is related to its cellular characteristics, such as cell size and number of neurons. Asymmetries in terms of cell size and density, regional volumes and the structure of dendrites have been reported by several authors^8–11^. The ratio of the volume of grey to white matter in cerebral hemispheres is 1:3 in young individuals and varies slightly and non-linearly with age (1:1 and 1:5 at 50 and 100 years old, respectively)^12^. In particular, the hemispheres differ in the number of neurons they contain: the left hemisphere has 186 million more neurons than the right hemisphere^13–15^.

Neuroimaging studies have suggested that asymmetries in water diffusivity in the brain may exist^16,17^. This asymmetry could be due to either a greater neuronal attenuation or a greater number of reciprocal connections with neighboring brain regions on the side with reduced diffusivity. Variations in water diffusivity in different areas of the brain change with age and with gender. Estrogens may affect diffusivity; they have been found to influence cerebral water diffusion by accelerating myelination in the immature rat brain^18^.

In this paper, we investigate the water dynamics in *post-mortem* sections of the right (RH) and left (LH) cerebral hemispheres of bovine specimens. The study takes advantage of the unique combination of the high water content of brain tissue (up to 80% in weight) and the power of neutron scattering to investigate water dynamics at the atomic scale^19,20^, thus extending the diffusion length-scales explored by the diffusion nuclear magnetic resonance (*d*MRI) technique and providing complementary information. In this way, we shed light on specific differences of the hemispheres which could at least partially be responsible for the various functions mentioned above.

## Results

### Interregional asymmetry in myelin structure

**Figure 1:**
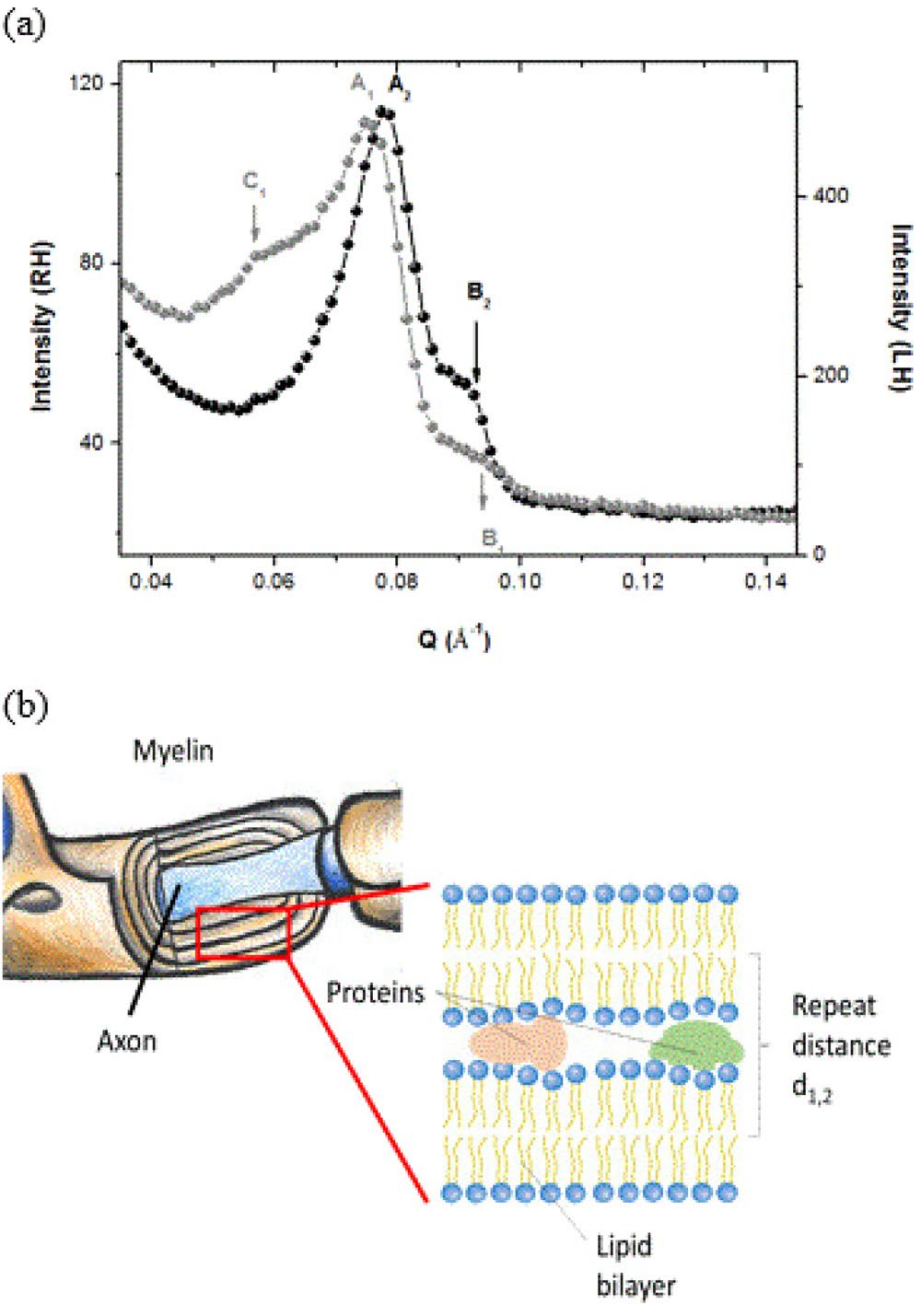
Panel a: Diffraction patterns obtained for the RH (black symbols) and LH (gray symbols) at 300 K measured on D16 at ILL. Second order Bragg peaks are found at A_1_ = 0.078 Å^−1^ (RH) and A_2_ = 0.074 Å^−1^ (LH). B_1_ and B_2_ represent less pronounced first (1^st^) or second (2^nd^) order Bragg peaks at Q = 0.093 Å^−1^ (RH) and Q = 0.088 Å^−1^ (LH). Additional reflection (C_1_) is observed in LH at Q = 0.057 Å^−1^. Panel b: Sketch of a myelin sheath and extraction of the multilamellar lipidic structure with attribution of the repeat distance d_1_ or d_2_.

In Fig. 1a, we present the neutron diffraction patterns of RH and LH specimens measured at 27 °C on the diffractometer D16 at the Institut Laue-Langevin (ILL) in Grenoble, France. Table 1 lists the d-spacing of the observed diffraction peaks (d). Diffraction peaks can be seen at Q = 0.078 Å^−1^ (A_1_) for RH and Q = 0.074 Å^−1^ (A_2_) for LH that correspond to the second order Bragg reflections of ordered multilamellar structures of the myelin sheath, with repeat distances of d_1_ = 161 Å (RH) and d_2_ = 170 Å (LH). These results are in agreement with previous findings^21–26^ from extracted myelin fibers. The first order Bragg peak is extinct by the form factor.

**Table 1:** Position of the Bragg reflections of RH and LH and associated repeat distances.

	RH		LH	
Bragg reflections	Q (Å^−1^)	d (Å)	Q (Å^−1^)	d (Å)
A	0.078	161	0.074	170
B	0.094	67 (1^st^)/134 (2^nd^)	0.091	69 (1^st^)/138 (2^nd^)
C	0.057	110 (1^st^)/220 (2^nd^)		

The arrows at B_1_ and B_2_ show the unresolved 1^st^ or 2^nd^ order Bragg peaks at Q = 0.092 Å^−1^ (d_1st_ = 134 Å, d_2nd_ = 67 Å) for RH and Q = 0.091 Å^−1^ (d_1st_ = 138 Å, d_2nd_ = 69 Å) for LH. The shift in the position of the Bragg reflections suggests different myelin arrangements in RH hemispheres compared to LH hemispheres, with a non-negligible change in the sheath thickness (9 Å). Moreover, an additional reflection is revealed in the diffraction pattern of LH (C_1_) at Q = Q = 0.057 Å^−1^ corresponding to a periodicity of d_1st/2nd_ = 110/220 Å. The origin of peaks B and C is not clear to date and more experiments need to be performed for this; thus they are not further treated in the present work. Figure 1b presents a sketch of the sheath arrangement.

### Interregional asymmetry in water dynamics at the micron to atomic scale

#### a) Micron scale

In conventional *d*MRI, the signal intensity, SI(b), in a free medium, is^27^:

1 $$SI(b)=S{I}_{0}{e}^{-bADC}$$

where b is the diffusion weighting factor, SI_0_ is the signal intensity at b = 0, and ADC is the apparent diffusion coefficient. The parameter *b* is linked to the magnitude of the applied diffusion gradient pulses, the duration of the pulses and the diffusion time.

**Figure 2:**
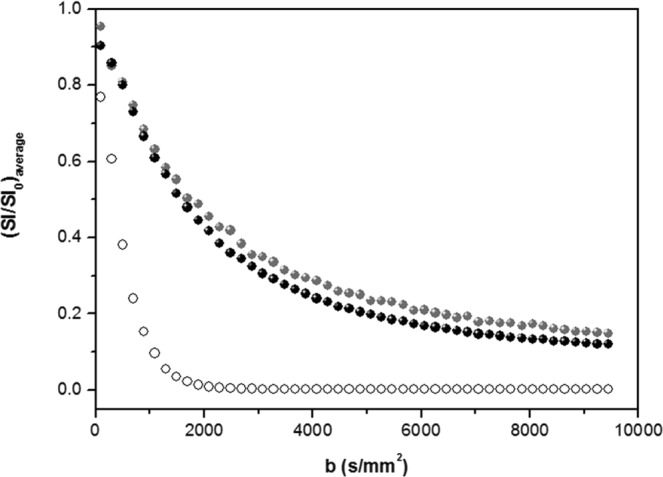
*d*MRI signal decay of RH (black symbols) and LH (gray symbols) averaged over the three orthogonal directions; for comparison, the averaged signal decay of free water (deionized water Milli-Q, open symbols) is also shown.

The signal decay SI (b) averaged (arithmetic mean) over the three orthogonal directions (x, y and z) is shown in Fig. 2, for RH and LH. For comparison, the signal of deionized water (Milli-Q) characteristic for pure water behavior is also reported, which suggests that a substantial fraction of the water in the brain has diffusive properties that are altered by the macromolecular environment.

We observe for RH and LH that the signal deviates from a mono-exponential decay, as would have been expected in an unrestricted and homogeneous medium with a single water population (represented here by the free water signal). Indeed water diffusion in the brain is not a free random walk process, but hindered by the crowded environment. We recently published a paper showing that the *d*MRI signal decay is better fitted by a double exponential, since the mono-exponential decay only fits in the low b-region, where the signal is less sensitive to confinement effects^28^.

We fit the data to the following expression for the double exponential function^29–31^:

2 $$\frac{SI(b)}{SI(0)}={f}_{slow}{e}^{-{D}_{slow}b}+(1-{f}_{slow}){e}^{-{D}_{fast}b}$$

where *f*_*fast *_= (1 – *f*_*slow*_) and *f*_*slow*_ represent the relative fractions of the fast and slow contributions to the total signal intensity with the associated fast (D_*fast*_) and slow (D_*slow*_) diffusion coefficients. The fit parameters are given in Table 2. A clear difference between diffusion coefficients is not found between the two hemispheres. However, the double-exponential analysis highlights the existence of different distributions of water populations in the two hemispheres: in LH the percentage of restricted water component (*f*_*slow*_) is greater compared to RH.

**Table 2:** Fitting parameters evaluated from the *d*MRI data using the bi-exponential model.

	*f*_*slow*_	D_*fast*_	D_*slow*_
RH	0.241 ± 0.007	0.610 ± 0.008	0.078 ± 0.003
LH	0.32 ± 0.01	0.62 ± 0.02	0.083 ± 0.004

D_fast_ and D_slow_ are the fast and slow diffusion coefficients and f_slow_ (with f_fast_ = 1- f_slow_) the fraction of the slow water pool. Units for D_fast_ and D_slow_ are expressed in [10^−5^ cm^2^/s].

#### Anisotropy

**Figure 3:**
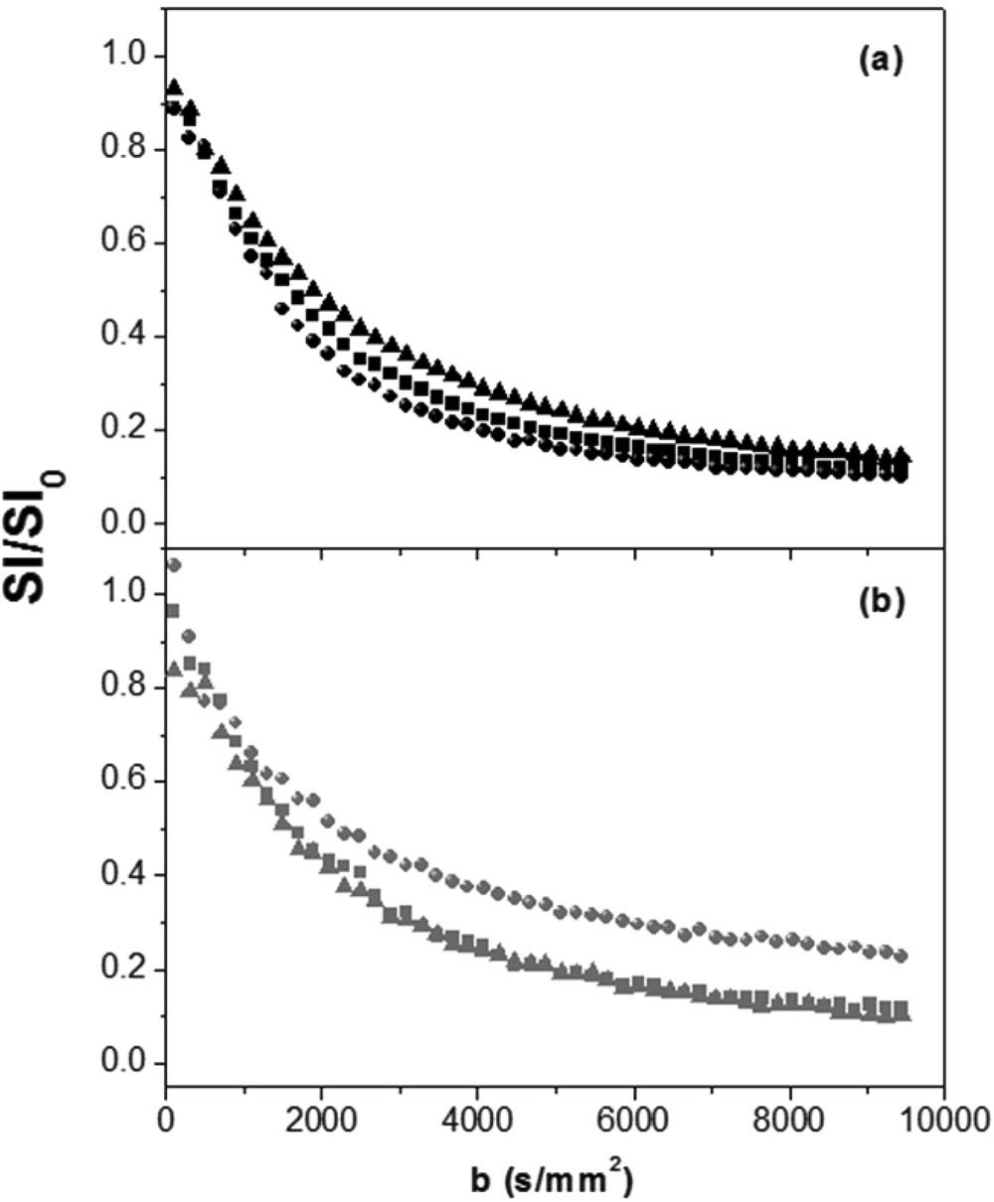
*d*MRI signal decay of RH (panel a) and LH (panel b) normalized to signal decay at b = 0.5 s.mm^−2^, for three orthogonal directions (x: squares, y: circles and z: triangles).

In Fig. 3, we present the dependence of the water signal on b measured with *d*MRI for the three orthogonal directions.

In the two samples, the water diffusion is clearly anisotropic, particularly in LH, in which one dimension (y) shows more restriction. The corresponding diffusion coefficients are reported in Table 3, where the *f*_*slow*_ percentage of LH(y) appears almost three times higher than that of RH(y).

**Table 3:** Fitting parameters evaluated from the *d*MRI data using the bi-exponential model as a function of the direction (x, y and z).

		*f*_*slow*_	D_*fast*_	D_*slow*_
RH	X	0.230 ± 0.006	0.605 ± 0.005	0.077 ± 0.003
	Y	0.17 ± 0.02	0.64 ± 0.02	0.055 ± 0.001
	Z	0.34 ± 0.01	0.59 ± 0.01	0.097 ± 0.004
LH	X	0.16 ± 0.03	0.51 ± 0.02	0.042 ± 0.002
	Y	0.46 ± 0.02	0.71 ± 0.03	0.074 ± 0.006
	Z	0.39 ± 0.04	0.77 ± 0.05	0.15 ± 0.01

D_fast_ and D_slow_ are the fast and slow diffusion coefficients and f_slow_ (with f_fast_ = 1 − f_slow_) the fraction of the slow water pool. Units for D_fast_ and D_slow_ are expressed in [10^−5^ cm^2^/s].

#### b) Atomic scale

**Figure 4:**
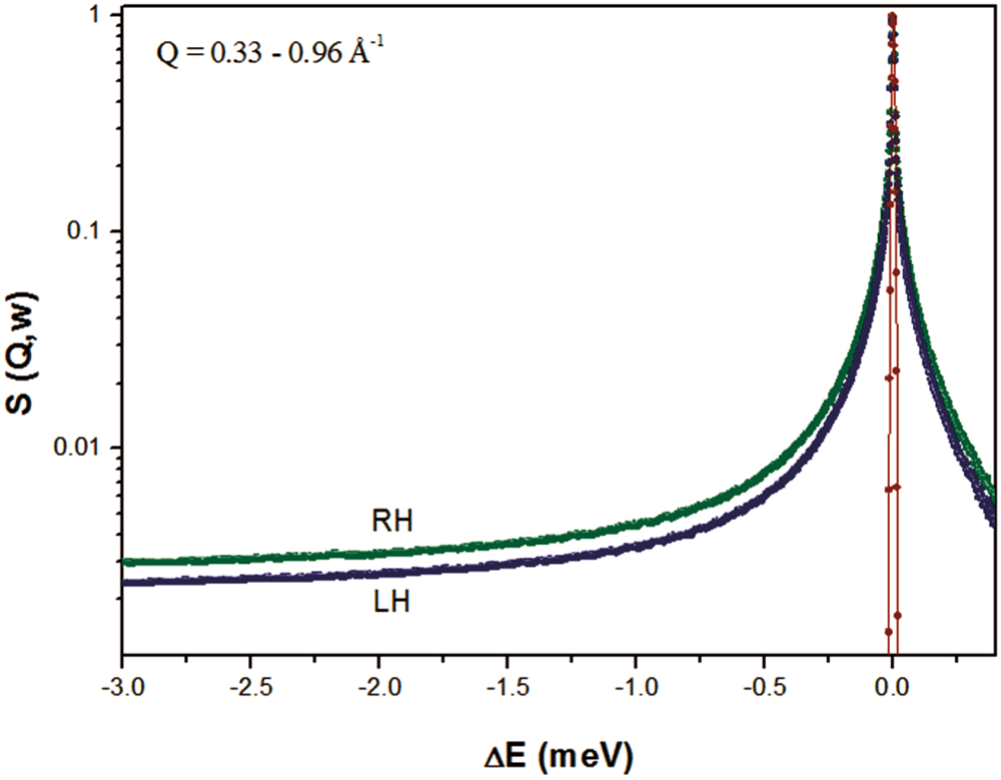
Q-binned QENS intensities measured on IN5 of RH (green) and LH (blue) at 300 K. For completeness, the instrumental resolution function is also shown (red).

In Fig. 4, we present the normalized incoherent quasi-elastic neutron scattering (QENS) spectra of RH and LH measured on IN5 as a function of the energy transfer, obtained by binning over the whole range of Q (0.33–0.96 Å^−1^). Data at Q ≤ 0.26 Å^−1^ were not included in the analysis because of the existence of Bragg peaks, as observed in the diffraction patterns reported in Fig. 1. In Fig. 4, an example of RH and LH QENS-fitted spectra at reference values of Q (Q = 0.40 Å^−1^ and 0.91 Å^−1^) is presented. For completeness, the instrumental energy resolution function is also shown. A broadening of the elastic peak can be observed, suggesting motion falling within the time scale accessible using IN5 (~100 ps). From the average composition of the RH and LH tissues (84% and 83% of water percentage in mass for LH and RH, respectively, as experimentally estimated as the mass change before and after drying the tissue), the incoherent scattering signal arising from the water has been theoretically estimated to be ~80% of the total intensity. Therefore, the main contribution to the QENS spectra is ascribed to water diffusion.

The Q-dependent QENS data were fitted using an equation which takes into account free and restricted water dynamics described in terms of a model based on the coupling between translational (T) and rotational (R) motions^32^. The translational model used is the jump diffusion model^33^ in which the diffusion is assumed to occur via infinitely small, elementary jumps characterized by a negligible jump time τ_j_ during which the particle diffuses and the residence time τ, i.e. the time a proton spends in a given position. The rotational model used is the continuous rotational diffusion on a circle^34^ describing the reorientation motion of a molecule, which rotates randomly on a spherical surface. An exhaustive description of the model and its application to the study of water dynamics in cells and tissues is reported in^19,20,28,35^. The fit function reads:

3 $$S(Q,\omega )\approx [\begin{array}{c}f\,\ast \,\delta (\omega )+{p}_{fast}\,\ast \,{S}_{fast}(Q,\,\omega ,\,{D}_{Tfast},\,{\tau }_{fast},\,{D}_{Rfast})+\\ {p}_{slow}\,\ast \,{S}_{slow}(Q,\,\omega ,\,{D}_{Tslow},\,{\tau }_{slow},\,{D}_{Rslow})+\\ {p}_{C{H}_{2}}\,\ast \,{S}_{C{H}_{2}}(Q,\,\omega ,\,{\varGamma }_{C{H}_{2}}(Q))\end{array}]\otimes R(Q,\,\omega )$$

where *f* is the elastic fraction, describing protons contributing only through atomic vibrations and whose center of mass appears stationary within the instrumental resolution, *R*(*Q*, *ω*) measured with a standard vanadium. *δ*(*ω*) is the Dirac delta function; *S*_*fast*_(*Q*, *ω*) and *S*_*slow*_(*Q*, *ω*) are the total scattering intensities arising from the bulk-like (fast) and restricted (slow) diffusive water populations, respectively. *D*_*Tfast/slow*_ and *D*_*Rfast/slow*_ are translational and rotational diffusion coefficients, and τ_fast/slow_ are the residence times. *S*_*CH2*_(Q, ω) is an additional term assigned to 2-site jump rotational motion (described by a large and Q independent Lorentzian of width *Γ*_*CH2*_) for the CH_2_ groups belonging to proteins and other cellular components and accounting for ~10% of the total signal. *p*_*fast*_, *p*_*slow*_ and *p*_*CH2*_ are the fractions of atoms experiencing the three different dynamics, with *f* + *p*_*CH2*_ + *p*_*fas*t_ + *p*_*slow*_ = 1.

The broadening of the Lorentzian scattering contributions (Γ_fast/slow_) while obeying the relation:

4 $$\,{\varGamma }_{\mathrm{fast}/\mathrm{slow}}\propto \frac{{D}_{\mathrm{Tfast}/\mathrm{slow}}{{\rm{Q}}}^{2}}{{\rm{1}}+{{\rm{\tau }}}_{\mathrm{fast}/\mathrm{slow}}{{\rm{D}}}_{\mathrm{Tfast}/\mathrm{slow}}{{\rm{Q}}}^{2}}$$

for the translational motion, is Q independent in the framework of the rotational one.

**Figure 5:**
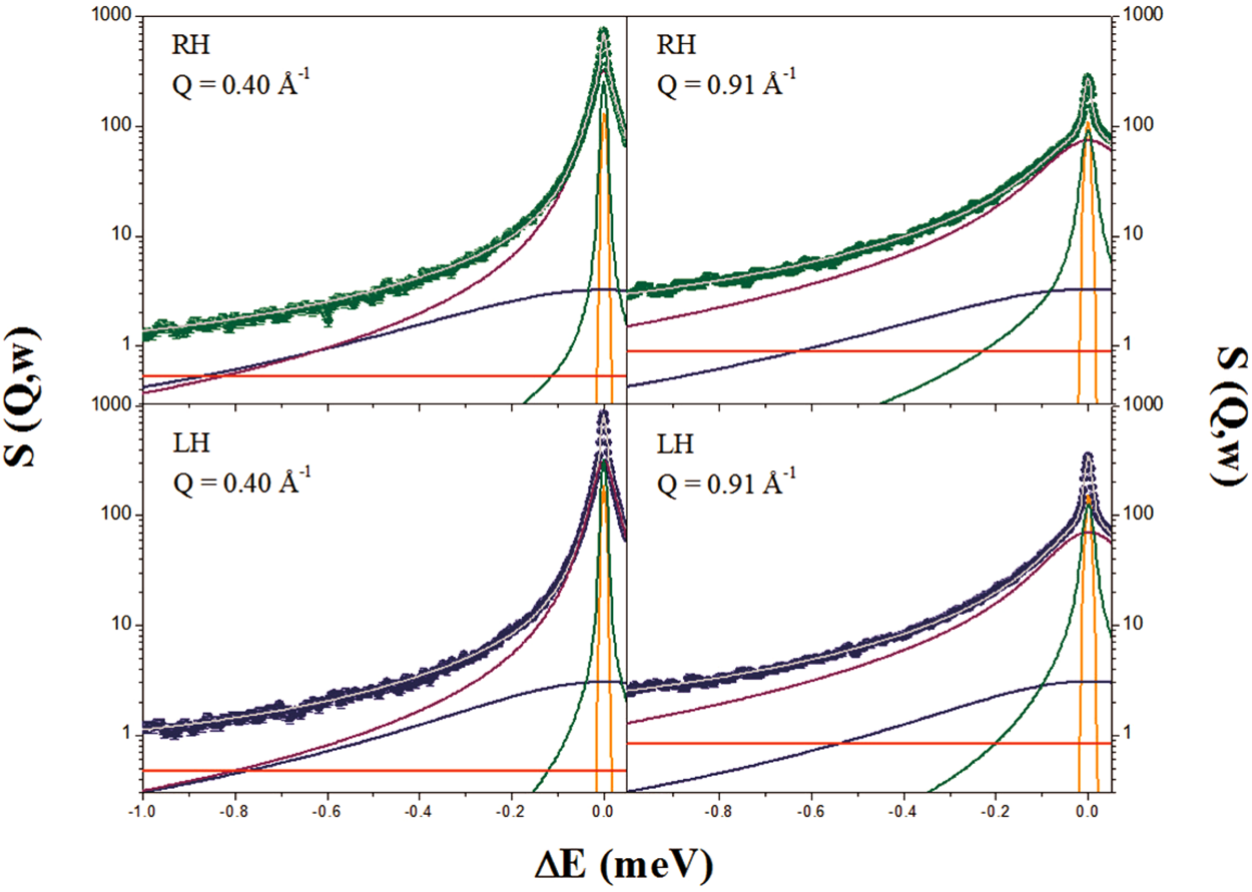
Example of QENS spectra of RH (green) and LH (blue) samples at Q = 0.40 Å^−1^ and Q = 0.91 Å^−1^ measured on IN5 at 300 K. The different scattering contributions, convoluted with instrumental resolution, are shown: experimental data (symbols), total fit (gray), elastic peak (orange), broad Lorentzian S_CH2_(Q, ω) (blue), fast water component (purple), slow water component (green) and background (red).

**Figure 6:**
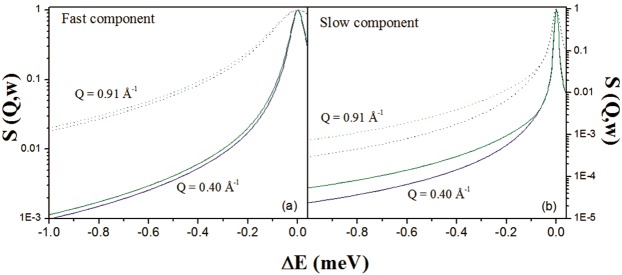
Contributions of the fast (panel a) and slow (panel b) water pool to the global QENS signal. RH: green; LH: blue; solid lines: Q = 0.40 Å^−1^; dotted lines: Q = 0.91 Å^−1^.

**Figure 7:**
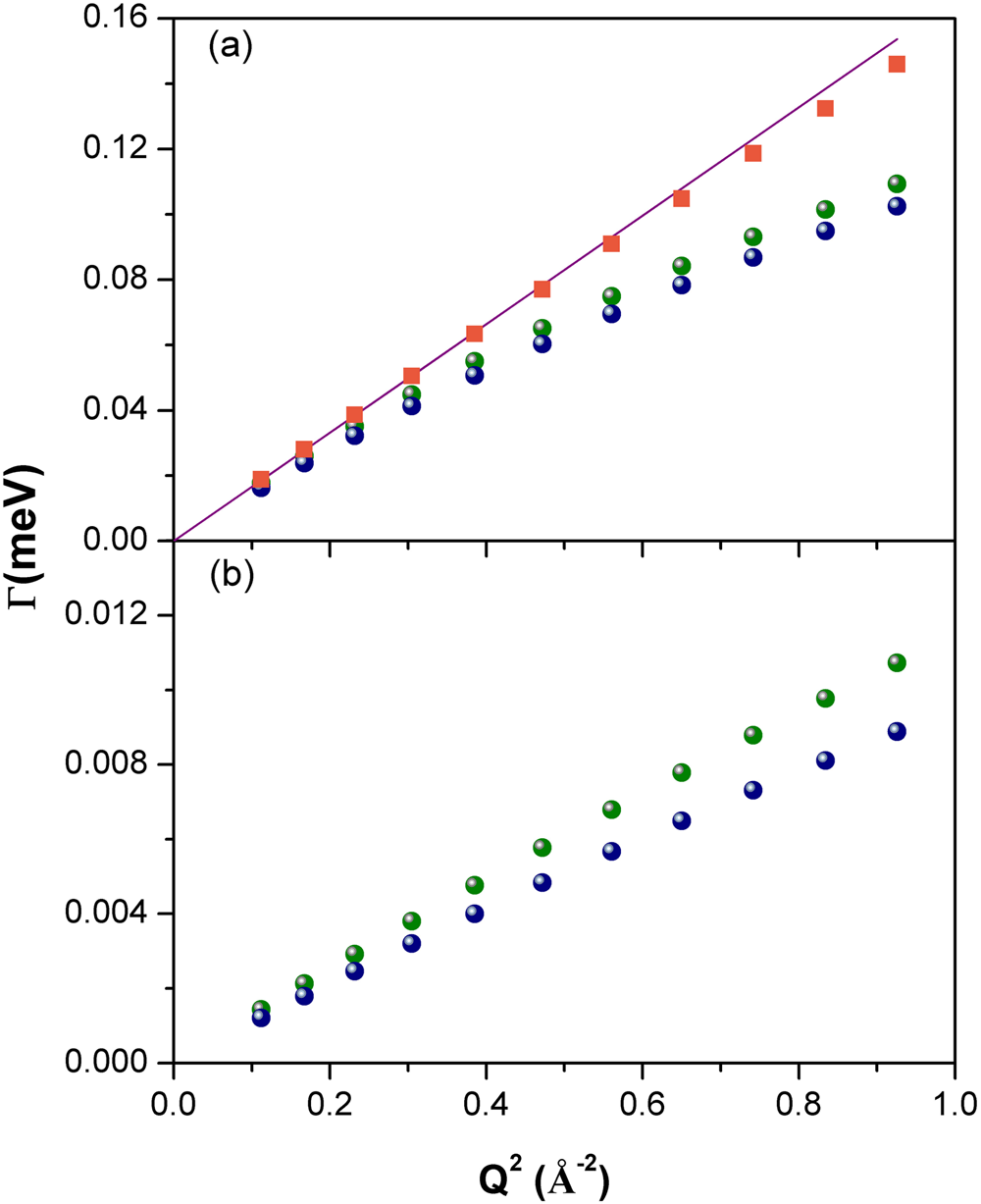
FWHM (Γ) of the translational motion for bulk-like water component (**a**) and restricted water component (**b**), for RH (green symbols), LH (blue symbols) and pure water (orange symbols), as a function of Q^2^, at 300 K. A Fick’s law function (purple line) has also been reported.

The measured spectra, in the Q-range from 0.33 to 0.96 Å^−1^, were fitted simultaneously from −3.0 to +0.1 meV using a global fit strategy. The different scattering contributions, convoluted with instrumental resolution, are shown. The agreement between experimental data (symbols) and theory (solid line) in Fig. 5 is excellent. Best-fit parameter values are reported in Table 4. It is clear that the main difference in terms of water dynamics in the two hemispheres lies in the diffusion of the restricted water pool. Both translational and rotational diffusions seem to be affected, as demonstrated by the changes in the residence time of the translational contribution and the rotational diffusion constants. For a direct comparison, in Fig. 6 the fast/slow components of RH and LH (convolution of translational and rotational diffusion with the instrumental resolution function) are shown. In Fig. 7, we report the variation of the line-width of the translational diffusive contribution as a function of Q^2^, at room temperature. Fick’s law function, describing a bulk-free Gaussian-like diffusion process, is also reported and, as expected, it properly fits the pure water data. For the two samples, after a linear variation in the low Q region (the translational diffusion coefficient is derived from the slope at small Q), the data deviate from Fick’s law, tending asymptotically to a constant value 1/τ_fast/slow_ (jump-diffusion model), with τ_fast/slow_ the residence time. The deviation at high Q from pure long-range diffusion is a sign of partial restriction, from which a non-zero residence time (the time that a proton spends in a given position) suggests the interaction of water molecules with the cellular components. This deviation from the theoretical behavior is the indication of non-Gaussian diffusion. The plateau becomes visible in the “free” water component (Q^2^ » 1/ Dτ and 1/D_fast_τ_fast_ ~ 2 Å^−2^), but this is less pronounced in the restricted water component (1/D_slow_τ_slow_ ~ 8 Å^−2^) even though a small deviation from Fick’s law is already noticeable. Again, the RH Γ values lie above the LH ones due to less restriction in this sample.

**Table 4:** Values of the parameters obtained when fitting at the same time 11 Q values, from 0.33 to 0.96 Å−1.

	*f*	*p*_*fast*_	*p*_*slow*_	*p*_*CH2*_	D_*Tfast*_	D_*Rfast*_	*τ*_*fast*_	D_*Tslow*_	D_*Rslow*_	*τ*_*slow*_	*Γ*_*CH2*_
RH	0.040 ± 0.002	0.736 ± 0.002	0.123 ± 0.002	0.100 ± 0.002	2.5 ± 0.1	2.3 ± 0.2	1.8 ± 0.1	0.20 ± 0.02	1.10 ± 0.05	6.7 ± 0.3	0.39 ± 0.02
LH	0.062 ± 0.003	0.715 ± 0.003	0.135 ± 0.003	0.088 ± 0.003	2.3 ± 0.2	2.3 ± 0.3	1.8 ± 0.5	0.16 ± 0.03	0.40 ± 0.03	9.7 ± 0.3	0.33 ± 0.05
Bulk water*					2.5	2.3	0.9				
Bulk water**					2.3		1.1				

*f* is the fraction of immobile protons; p_fast_, p_slow_ and p_CH2_ are respectively the fraction of free water population, of restricted water population and of protons not attributed to water. D_Tfast/slow_, D_Rfast/slow_ and τ_fast/slow_ are the translational and rotational diffusion coefficients and residence times of free water (fast) and restricted water (slow). Γ_CH2_ is the line-width of the S_CH2_ (Q, ω) contribution. Units for D_Tfast_, D_Tslow_, D_Rfast_, and D_Rslow_ are expressed in [10^−5^ cm^2^/s], while τ_fast_ and τ_low_ are expressed in [ps] and Γ_CH2_ in [meV]. * = Diffusion coefficient of bulk water determined experimentally from a scan of pure water measured at 300 K on IN5 at the same energy resolution. ** = Diffusion coefficient of bulk water found in literature^42,47^ for T = 298 K. The associated errors were estimated using the Minuit minimization algorithm and the Minuit processor MINOS^60^.

## Discussion

Lateralized behaviors (e.g. limb or eye preference) have been observed in a wide range of species, including dairy cows^36,37^. In humans, it is well accepted that there are several lateralization axes in the brain (symbolic communication, perception/action, emotion…)^38^. Moreover, lateralization abnormalities have been observed in patients with schizophrenia and bipolar disorders^39^.

Using electron microscopy, differences in the axonal diameter and in the thickness of myelin sheath of the cortical white matter were observed in humans and macaque^40^. These differences lead to change in conductivity between left and right hemispheres. The benefit of such difference is not clear yet, but it has recently been proposed that it could lead a hemispheric dominance associated to a reduced inter-hemispheric communication and thereby improve processing time of lateralized functions^38^.

Therefore, lateralization seems to have important effects on the correct functioning of the brain, although studies of it are still scarce. The powerful combination of complementary *d*MRI and QENS experiments on RH and LH allows to investigate variations at the molecular dynamics’ level to reveal the non-Gaussian behavior of water dynamics at both micron and atomic scales. In particular, two different water components are characterized and quantified: a population of water hindered by macromolecular surfaces and not freely diffusing, and another population with a behavior similar to free water. In particular, the QENS analysis reveals that a major fraction (86% ± 1% for RH and 84% ± 2% for LH, percentage relative to total water) demonstrates fast dynamical properties similar to those of bulk water, and a minor fraction (14% ± 3% RH and 16% ± 4% LH) displays significantly slower dynamics. There is no significant difference between the percentages of free and restricted components in the two hemispheres, suggesting comparable samples.

For the bulk-like water contribution, translational diffusion coefficients for the two samples are found to be similar to those of bulk water (2.5 ± 0.1 ·10^−5^ cm^2^/s in RH and 2.3 ± 0.1 * 10^−5^ cm^2^/s in LH; 2.3 ÷ 2.5 * 10^−5^ cm^2^/s bulk water)^36^ but with higher residence times for proton jump diffusion (1.8 ps in RH and 1.8 ps in LH, 0.90-1.1 ps in bulk water). The higher residence times may reflect the longer times spent by the protons interacting with heterogeneities in the internal cerebral structure. This is in agreement with findings that have been observed in *Escherichia coli* (*E*. *coli*)^41,42^, and red blood cells (RBC)^43^, prokaryotes^44^ and lipoproteins^45^. In particular, the higher residence times observed in *E*. *coli* and RBC have been interpreted by suggesting that water molecules spend longer times in the first hydration shell of macromolecular structures than in the bulk phase. Jasnin *et al*. found moreover that a higher residence time is related to interactions between molecules and to exchange mechanisms of biomolecules with hydration water^46^.

On the other hand, the rotational diffusion coefficients for the bulk-like water component, for the two samples, are in agreement with the value for bulk water (2.3 10^−5^ cm^2^/s)^47^.

In conclusion, the bulk-like water population has diffusion rates typical for pure free water but with a different residence time. However, no difference is found for this component between RH and LH.

The translational and rotational diffusive contributions of the restricted water proton dynamics show non-negligible variations from bulk water, confirming a strong reduction in water mobility. In particular, the translational diffusion coefficients (0.20 ± 0.02 10^−5^ cm^2^/s in RH, 0.16 ± 0.04 10^−5^ cm^2^/s in LH) are one order of magnitude lower than those for bulk water and they are hemisphere-independent. On the other hand, a higher residence time is found for LH compared to RH. In the rotational diffusion coefficient (1.1 ± 0.1 10^−5^ cm^2^/s in RH and 0.40 ± 0.06 10^−5^ cm^2^/s in LH), strong hemisphere dependence is also found. This difference highlights the suppressed water dynamics in LH. In short, a second population of water with restricted behavior is found, with properties, which differ from one hemisphere to the other, resulting, in particular, in suppressed dynamics in LH.

The results obtained with *d*MRI for the diffusion coefficient (0.078 ± 0.003 10^−5^ cm^2^/s in RH and 0.083 ± 0.004 10^−5^ cm^2^/s in LH) are in accordance with the behavior of water diffusion shown with neutron spectroscopy, even though they are an order of magnitude different from those found in QENS measurements. The time over which the *d*MRI measurements are made is sufficiently long that significant interaction of water molecules with cellular constituents occurs. Indeed, during the 11 ms of diffusing time (t_d_) applied here, the water molecules are able to explore a distance (x) of ~4 μm (x^2^ = 2·D·t_d_, with D the water diffusion coefficient at room temperature), i.e. the typical size of a cell (from 0.9 μm for dendrites to 20–30 mm for ribosomes). Thus, viscosity and macromolecular crowding are among the possible causes of the reduction of the diffusion coefficients^28,48,49^. On the other hand, obstructive effects less influence D measured by neutron scattering, being a probe of atomic scale at which the macromolecular separation occurs. As revealed by our diffraction experiments (Fig. 1a), the compaction of the myelin sheath is clearly different in the two hemispheres. So the different microscopic structure between the two hemispheres may explain the differences in water diffusion. The enhanced anisotropic behavior of LH shown in Fig. 3 also supports this idea. Indeed, the restriction may not be the same for different directions of motion. In a study by Denis Le Bihan *et al*.^50^, the diffusion coefficient was found to be significantly smaller when the gradient pulses of the diffusion imaging sequence are perpendicular to the myelin fiber direction. The diffusion of water is restricted differently along and across fibers: in the direction transverse to the fibers, water motion is prevented by the presence of the myelin sheet. This is in agreement with the earlier findings of Anderson *et al*.^51^, who reported interregional asymmetries in myelin sheath thicknesses through electron microscopy on *post-mortem* posterior superior temporal lobes in humans. In particular, left hemispheres were seen to be characterized by larger diameter axons surrounded by a thicker myelin sheath, reflecting the faster conduction required by the rapid sensory signal processing performed by the left hemisphere. To the best of our knowledge, the investigation of structural asymmetries of myelin size has not been extended, up to now, to other cortical areas. Thus, our work provides unique experimental evidence reinforcing the idea of left-to-right asymmetry in hemisphere tissue myelination. Unfortunately, the anisotropic behavior cannot be investigated at the atomic scale, since QENS provides averaged information on the dynamics of all the protons constituting the sample. In order to be direction sensitive, QENS scans must be acquired on oriented samples such as myelin fibers.

Moreover, Penhune and Hervé^52,53^ highlighted interregional asymmetry in the primary auditory areas^13^, which they suggested was due to the existence of larger volumes of white matter in the LH and thus to differences in the cellular organization of the two hemispheres, as speculated by Kantarci K. *et al*.^54^, who proposed that neuron and glia were more compactly packed in LH than in RH. Similar findings were reported by Amunts *et al*.^55^, who showed that the right hemisphere was characterized by a greater percentage of cell soma in the cortical regions (difference in volume density of nerve cells up to 5%), while the left hemisphere contained more dendrites, axons and synapses.

The diameter of the fibers and the thickness of the myelin sheath may be small (e.g. as thin as 0.1 µm^56^,) and thus challenging to address with *in vivo d*MRI, a technique which has a low sensitivity to short diffusion times^57^. Probing water diffusion at the atomic scale using neutron scattering could therefore help describing fine differences in brain conductivity and help understand how brain lateralization contributes to brain function. Further experiments are required to evaluate how changes in myelin sheath properties and/or axonal diameter can be evaluated by neutron scattering.

## Materials and Methods

### Tissue extraction and sample preparation

Fresh post-mortem bovine brains were obtained from the slaughterhouse in Padova (Italy). Brain tissues were extracted at the Department of Animal Medicine, Production and Health of the University of Padova (Italy). The brains were removed in two parts: the cerebellum and the cerebrum, then separated at the junction of the pons and the cerebral peduncle. Sections (0.5 × 1 cm^2^) of RH and LH were extracted from the cerebrum and frozen at −160 °C in liquid nitrogen vapor and divided in two prior to the *d*MRI and neutron scattering experiments.

### *d*MRI experiments

*d*MRI measurements of bovine brain tissue at room temperature were performed at the MRI Facility of Grenoble (IRMaGe, France) using a 4.7 T Biospec 47/40 USR system (Bruker, Ettlingen, Germany), capable of delivering gradient strengths of 600 mT/m in 120 μs, and a volume transmit/receive coil. T2 (transverse relaxation time) control scans were performed using a Carr Purcell Meiboom Gill sequence (64 spin-echoes between 5 and 320 ms, TR = 2000 ms, field of view = 70 × 70 mm², matrix = 128 × 96, slice thickness = 3 mm, 2 averages). Diffusion weighted images were obtained using a spin-echo echo-planar sequence (TE = 23.2 ms, δ = 7 ms, Td = 11 ms, TR = 2000 ms, same geometry as that of the T2 sequence above, matrix = 64 × 48, 2 averages). Twelve reference images (b = 0.5 s/mm², where b is the diffusion weighting factor) were followed by diffusion weighted images. Forty-eight values of b, varying between 100 and 9400 s/mm², were applied in each of the 3 gradient orientations (x, y and z with a total of 144 diffusion weighted images). Data were further analyzed using a bi-exponential model^29–31^.

### Neutron scattering experiments

For the neutron experiments, 50 μm thick slices of RH and LH were cut using a cryotome (Thermo Scientific, Shandon Cryotome SME Cryostat, 77210160 - France) at the histological laboratory of the Biomedical Facility of the European Synchrotron Radiation Facility (ESRF, Grenoble-France), while preserving the tissue temperature at constant −20 °C. The thickness of the tissue slices was chosen in order to limit neutron absorption from water (H_2_O), thus minimizing multiple scattering events. The sample was closed in vacuum-tight pure aluminum flat sample holder.

For the diffraction experiments, to enhance the coherent signal and the neutron contrast, the tissue was left to equilibrate in a D_2_O atmosphere for 30 minutes in order to promote isotope H-D exchange. The coherent scattering cross-section of D is indeed larger with respect to H (σc_D_ = 5.6 10^-24^ cm^2^; σc_H_ = 1.76 10^-24^ cm^2^). The resulting diffraction pattern was obtained on the small momentum transfer diffractometer D16 ILL (Grenoble, France). The instrument was operating with the MILAND ^3^He position sensitive detector consisting of 320 × 320 detection channels with 1 mm resolution, resulting in a sensitive area of 32 × 32 cm^2^. The monochromator was set to deliver a wavelength λ = 4.75 Å with Δλ/λ = 0.01 (FWHM) and the sample-to-detector distance was set to 950 mm. The data were corrected for detector efficiency, normalized to incident neutron flux and integrated vertically in the range −1.2° < 2θ_vert_ < 2.8°.

To estimate the diffusion coefficient of protons at the atomic scale, QENS experiments were carried out at room temperature on the high-resolution time–of–flight spectrometer IN5^58^ at ILL.

For the present work, we chose the instrument setup configuration at λ = 10 Å, corresponding to an energy resolution of δE ~ 10 µeV FWHM (which is equivalent to 10^−10^ s) and Q < 1.1 Å^−1^ (equivalent to 6 Å). Consecutive short QENS scans were acquired (15 minutes each) and compared before binning. During the measured laps of time (2 hours), the scattering signal was stable, meaning that no variation was observed in proton dynamics. Data reproducibility and changes in the tissues dynamics upon the conservation protocol, cryogenic towards formalin addition, were discussed in dedicated papers published by our group^19,20^.

The program LAMP^59^ was used for data reduction, consisting in the a) normalization of the raw data to the neutron flux, b) subtraction of the empty cell contribution and c) normalization with respect to a vanadium scan (a totally incoherent sample) to compensate for differences in detector efficiency and geometry. In order to avoid corrections from multiple scattering events, cell thickness were properly chosen to minimize neutron absorption from the sample (sample transmission ∼90%).

## Conclusion

Our data showed that significant differences in the behavior of water exist between RH and LH in bovine brain hemispheres, with the water in LH more restricted than in RH both in the micron and atomic scales, accompanied by asymmetries in the myelin sheath thickness. Although the composition of such physiological systems is complex, the average proton dynamics at the atomic scale (ps-ns time scale) probed using QENS reveals significant differences in water diffusion when looking at tissues. The QENS analysis allowed us to quantify and discriminate free and restricted water diffusion processes through translational and rotational diffusion coefficients and residence times. Interregional asymmetries are found in the dynamic properties of the restricted water pool, while the bulk-like component remains unaffected. Moreover, our *d*MRI measurements allowed us to highlight the anisotropic behavior of water diffusion within the tissue.

## Data Availability

The data are accessible at osf.io/adw9q. Data Digital Object Identifier: https://doi.ill.fr/10.5291/ILL-DATA.8-05-416.
